# Clozapine response in patients with treatment-resistant schizophrenia: a preliminary report

**DOI:** 10.1192/j.eurpsy.2025.2090

**Published:** 2025-08-26

**Authors:** Z. Maridaki, L. Mantonakis, E. Vasilopoulos, N. Smyrnis, N. Stefanis, N. Kokras, E. Tzavellas

**Affiliations:** 1 1st department of Psychiatry, Eginitio Hospital, National and Kapodistrian University of Athens; 2 2nd Department of Psychiatry, “ATTIKON” University General Hospital, National and Kapodistrian University of Athens, Athens, Greece

## Abstract

**Introduction:**

Clozapine, the first atypical antipsychotic agent, serves as the gold standard treatment for treatment-resistant schizophrenia (TRS), being the most effective choice. Despite the fact that early commencement of clozapine is related to a higher response rate of patients, many psychiatrists remain reluctant towards its initiation. Identifying sociodemographic and clinical features correlating to clozapine response could facilitate the prompt clozapine initiation and ameliorate clinical outcomes.

**Objectives:**

Our department presents the preliminary results of a prospective cohort study on patients diagnosed with TRS, as defined by the Treatment Response and Resistance in Psychosis (TRRIP) Working Group criteria, before clozapine initiation prospectively for 6 months. The study aims to evaluate the potential association of several sociodemographic and clinical factors with clozapine response.

**Methods:**

The patients included in our study were required to have a history of treatment-resistant schizophrenia, as specified by the TRRIP criteria and no history of treatment with clozapine. The TRS patients were submitted to clinical assessments in baseline and 6 months after clozapine initiation included the following: the Positive and Negative Syndrome Scale (PANSS), the Perrsonal and Social Performance Scale (PSP), past medical history, sociodemographic data, blood tests, and monitoring of clozapine blood levels.

**Results:**

30 patients have been screened until now, of which, 26 met the inclusion criteria. 4 patients withdrew from the study before the 6-month follow-up, 4 patients discontinued clozapine due to serious adverse events. 18 patients successfully completed the follow-up. 50% of the TRS patients showed significant clinical response to clozapine, as described by >20% increase in PANSS score. Clozapine responders also showed a significant increase in functionality, as assessed by the elevation of the PSP score (p <0,001), as expected. However, neither PSP score nor PANSS positive, negative or total score at baseline were predictive of clozapine response. Regarding the patients’ sociodemographic data, no statistically significant differences were identified between clozapine responders and non-responders.This study is also in accordance with the existing literature suggesting a significant delay in clozapine prescription by physicians. In our study, 80% of patients were prescribed more than three different antipsychotics before clozapine was initiated.

**Conclusions:**

Clozapine is an effective treatment for TRS, as supported by the preliminary results of our study. 50% of the TRS patients showed significant clinical response to clozapine, as shown by reduction in PANSS score, and increase in PSP score, as a measure of functionality. However, larger clinical samples are needed to showcase further, more delicate differences among the two groups, to highlight potential predictive factors of clozapine response.

**Disclosure of Interest:**

None Declared

